# Advancement of Physical and Photoelectrochemical Properties of Nanostructured CdS Thin Films toward Optoelectronic Applications

**DOI:** 10.3390/nano13111764

**Published:** 2023-05-30

**Authors:** Walid Ismail, Ghada Ibrahim, Mohamed A. Habib, Omar K. Alduaij, Mahmoud Abdelfatah, Abdelhamid El-Shaer

**Affiliations:** 1 Physics Department, Faculty of Science, Kafrelsheikh University, Kafrelsheikh 33516, Egypt; walid_s2@yahoo.com (W.I.); elshaer@sci.kfs.edu.eg (A.E.-S.); 2 Department of Chemistry, College of Science, Imam Mohammad Ibn Saud Islamic University (IMSIU), Riyadh 11623, Saudi Arabia; okduaij@imamu.edu.sa; 3 Chemistry of Tanning Materials and Leather Technology Department, Chemical Industries Institutes, National Research Center, Dokki, Giza 12622, Egypt

**Keywords:** nanostructured CdS thin films, hydrothermal method, electrochemical impedance spectroscopy (EIS), Mott–Schottky measurements, optoelectronic applications

## Abstract

CdS thin films were grown on an FTO substrate at different temperatures, employing the low-cost hydrothermal method. All the fabricated CdS thin films were studied using XRD, Raman spectroscopy, SEM, PL spectroscopy, a UV–Vis spectrophotometer, photocurrent, Electrochemical Impedance Spectroscopy (EIS), and Mott–Schottky measurements. According to the XRD results, all the CdS thin films were formed in a cubic (zinc blende) structure with a favorable (111) orientation at various temperatures. The Scherrer equation was used to determine the crystal size of the CdS thin films, which varied from 25 to 40 nm. The SEM results indicated that the morphology of thin films seems to be dense, uniform, and tightly attached to the substrates. PL measurements showed the typical green and red emission peaks of CdS films at 520 nm and 705 nm, and these are attributable to free-carrier recombination and sulfur vacancies or cadmium vacancies, respectively. The optical absorption edge of the thin films was positioned between 500 and 517 nm which related to the CdS band gap. For the fabricated thin films, the estimated Eg was found to be between 2.50 and 2.39 eV. According to the photocurrent measurements, the CdS thin films grown were n-type semiconductors. As indicated by EIS, resistivity to charge transfer (RCT) decreased with temperature, reaching its lowest level at 250 °C. Flat band potential and donor density were found to fluctuate with temperature, from 0.39 to 0.76 V and 4.41 × 10^18^ to 15.86 × 10^18^ cm^−3^, respectively, according to Mott–Schottky measurements. Our results indicate that CdS thin films are promising candidates for optoelectronic applications.

## 1. Introduction

Cadmium sulfide (CdS) is one of the prominent members of the II–VI semiconductor group due to its innovative technological and physical characteristics, and is also used in industry [1]. CdS is an n-type semiconductor material due to its intrinsic donor imperfections such as S vacancies and Cd interstitials [2], and has a wide direct band gap of approximately 2.42 eV [3]. CdS thin films have a high mobility (0.1–10 cm^2^/v·s), a high absorption coefficient (10^4^–10^5^ cm^−1^), a high refractive index, and high electrochemical stability [4]. CdS thin films have very high photosensitivity in the visible region and have been employed increasingly frequently in solar cells as window layers [5], photodetectors [6], transistors [7], light-emitting diodes (LEDs) [8], optoelectronic devices [9], photoconductive sensors, and photonic devices [10]. CdS has two crystal structures, a metastable cubic phase (zinc blende) and a highly stable hexagonal phase (wurtzite), where thin films can be formed during these two phases based on growth conditions [11,12]. CdS thin films were deposited using several synthesis techniques such as spray pyrolysis [13], sputtering [14], hydrothermal method [12], chemical bath deposition (CBD) [15], thermal evaporation [11,16], chemical vapor deposition (CVD) [17], electrodeposition [18], and sol-gel deposition [19]. The hydrothermal technique has many advantages for growing CdS thin films, including low-cost, environment, high purity, compositional consistency of the thin film, and the simplicity of the method [20]. The carrier’s effective mass and the iconicity of the CdS are both high. Additionally, it can operate with short radiative carrier lifetimes and short carrier dispersion lengths [21]. These important characteristics make CdS a suitable choice for the manufacture of optoelectronic devices and the improvement of the window layer in contemporary solar cells. The main factors affecting the optical and electrical characteristics of CdS nanostructures, as well as their applications, are their size, shape, and/or morphology [22]. Such properties can vary depending on growth conditions such as precursor concentrations, deposition time, solution temperature, solution pH, and substrate type. A. Oliva et al. and N. S. Kozhevnikova et al. investigated the early stages of CdS thin film deposition on various substrates [23], and the principle formation of nanocrystalline CdS thin films [24]. The results showed that the formation of a cadmium hydroxide barrier layer between the CdS thin film and the Si substrate is required for the production of an adhesive CdS thin film. Temperature is considered to be one of the most important parameters in controlling the properties of CdS thin films. Therefore, CdS thin films were grown on FTO substrates employing the hydrothermal technique with preparation temperatures which varied from 50 to 250 °C. Various characterization techniques were used to specifically investigate the effect of temperature on the morphological, microstructure, optical, and photoelectrochemical properties of CdS thin films.

## 2. Materials and Methods

The hydrothermal technique was applied to fabricate CdS thin films on fluorine-doped tine oxide (FTO) glass substrates. The FTO substrates were washed with acetone, ethanol, and deionized water (DIW) (Milli-Q, 18 MΩ cm) using ultrasonic cleaner, and then dried in an oven at 100 °C for 30 min. The substances in the chemical solution utilized were 0.8 M cadmium sulfate (CdSO_4_·8H_2_O; 98%, BDH, UK) and 0.08 M thiourea (CS (NH_2_)_2_; 99%, NICE, Cochin, Kerala) [22]. The precursors were dissolved in 20 mL of DIW in separate beakers, and then the thiourea solution was added to the cadmium sulfate solution and mixed completely at room temperature for 20 min by rapidly stirring with a magnetic stirrer. The ammonia solution ((NH_3_); 32%, PIOCHEM, Egypt) served as a complex agent to adjust the pH value to 10. For the hydrothermal procedure, the resultant mixture was placed into a Teflon container with FTO substrate, and the container was then sealed and placed in a autoclave made of stainless steel for 2 h at 50 °C. After the period of preparation, the autoclave was allowed to cool naturally to ambient temperature. The CdS thin film was cleaned ultrasonically with deionized water. This procedure was repeated at different temperatures (from 50 up to 250 °C).

X-ray diffraction (XRD-6000 Shimadzu, Kafrelsheikh University) and scanning electron microscopy (SEM) (JSM-651OLV, Kafrelsheikh University) were utilized to examine the morphological and structural characteristics of the fabricated thin films. The optical characteristics of the thin films were examined using a UV–Vis spectrophotometer (JASCO V-750), PL was examined with a 3.82 eV (325 nm) Kimmon He-Cd laser, and the spectra were recorded with a built-in Synapse CCD camera on a HORIBA iHR320 spectrometer. A WITec alpha300 R system was employed so Raman spectra could be used. The photocurrent was measured using a self-made system that consisted of a light source (a 200 W tungsten lamp), an illumination switch (a manually operated light chopper with predetermined time intervals of 6 s), a three-electrode cell, and a controlling Bio-logic system. Photocurrent measurements were made at 0 Volts against an electrode of Ag/AgCl with a supporting electrolyte of 0.5 M Na_2_SO_4_. A A CHI660E electrochemical workstation was used to perform Electrochemical Impedance Spectroscopy (EIS) and Mott–Schottky measurements.

## 3. Results and Discussion

### 3.1. Structural Analysis

**Figure 1:**
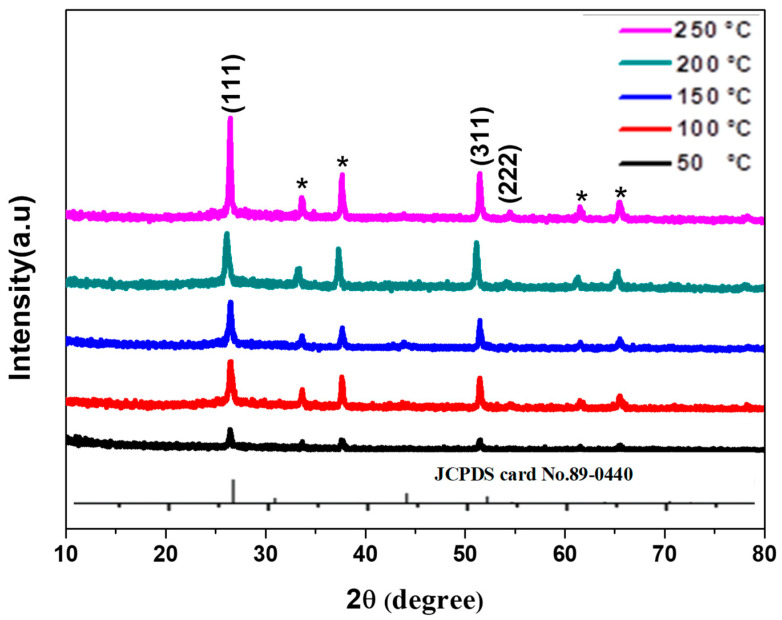
XRD pattern of deposited CdS thin films with various temperatures (* refers to FTO).

Figure 1 shows the XRD patterns of CdS thin films which were synthesized at various temperatures using hydrothermal techniques. The CdS films were grown in a cubic phase structure indexed with the reference card JCPDS No.89-0440 [25]. The diffraction angles were 26.4° and 51.4° which correspond to planes (111) and (311), respectively. Plane (111) was the preferable growth orientation. Other diffraction peaks of FTO substrates which indicate thinner films [26] appeared. The intensity of the main diffraction peaks increases proportionally as the temperature rises from 50 °C to 250 °C. No other phases appeared, indicating the high quality of the fabricated thin films. The crystallite size (*D*) of the fabricated CdS thin films increased as the temperature of preparation increased, something which was determined by the Scherrer equation shown in Equation (1) [27]:

(1) $$D=\frac{\;0.9\lambda }{\beta cos\theta }\;$$

where *λ* is the wavelength of the Cu K_α_ radiation source (1.54060 Å), β represents full width at half maximum in radians (FWHM), and θ  is the angle of diffraction. By changing the temperature of preparation, the crystallite size values fell between 25 and 40 nm. Williamson and Smallman’s relations were used to estimate the dislocation densities (*δ*) of the deposited films according to Equation (2) [28] and the crystal lattice strain $\left(\varepsilon \right)$ was determined by Equation (3) [29].

(2) $$\delta =\frac{1}{D^{2}}$$

(3) $$\;\;\;\;\;\;\;\;\;\;\;\;\;\varepsilon =\frac{\beta }{4\tan\theta }$$

**Figure 2:**
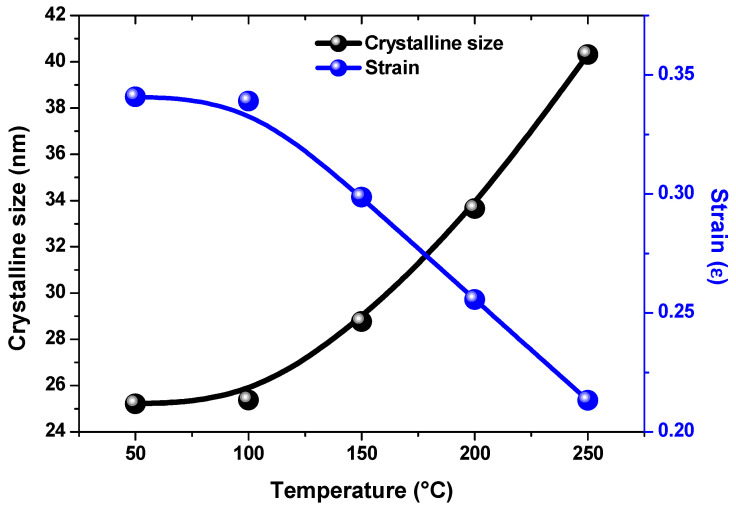
Variation in strain (*ε*) and crystalline size for hydrothermal CdS thin films.

The calculated values for *D*, *δ*, and ε are listed in Table 1. The microstrain and dislocation densities decreased with rising temperature as shown in Figure 2. This decrease represented a smaller number of lattice defects and high-quality thin film formation. This could be attributed to a reduction in the incidence of grain boundaries that comes from a rise in the crystallite size of thin films with increasing temperature [30,31].

### 3.2. Raman Spectroscopy Analysis

**Figure 3:**
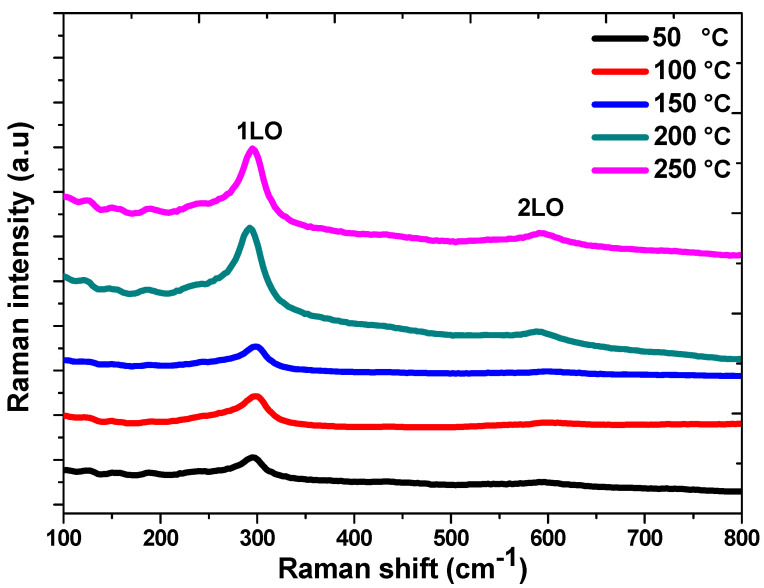
Raman spectra of CdS samples at different deposition temperatures.

The structure and electronic characteristics of the materials were measured using Raman spectroscopy [32]. Figure 3 shows the Raman spectra of CdS films synthesized at various temperatures. Each Raman spectrum has a prominent peak at 295.5 cm^−1^ which is assigned to first order (1LO) and a weak peak at 593.9 cm^−1^ which is attributed to second-order longitudinal optical (2LO) [32,33,34] for CdS. Raman spectroscopy thus provides additional evidence for the formation of CdS films in a cubic structure. It has been observed that the intensity of the 1LO Raman peak increases with rising temperature; this might be related to the growing size of the CdS grains [35]. These results show good agreement with the measurements of XRD.

### 3.3. Surface Morphology Analysis

**Figure 4:**
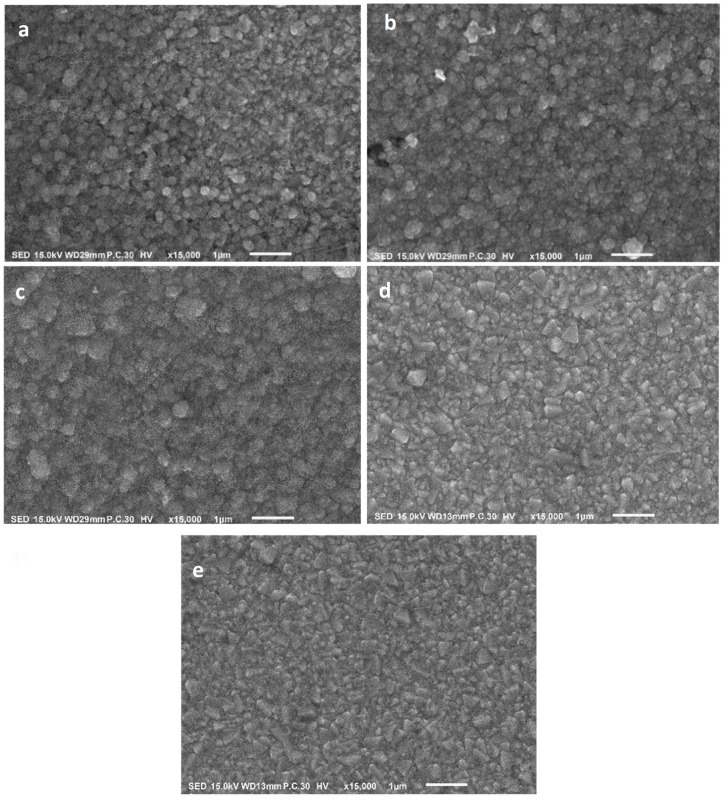
Top-view SEM images of CdS films prepared at (**a**) 50 °C (**b**) 100 °C (**c**) 150 °C (**d**) 200 °C, and (**e**) 250 °C.

**Figure 5:**
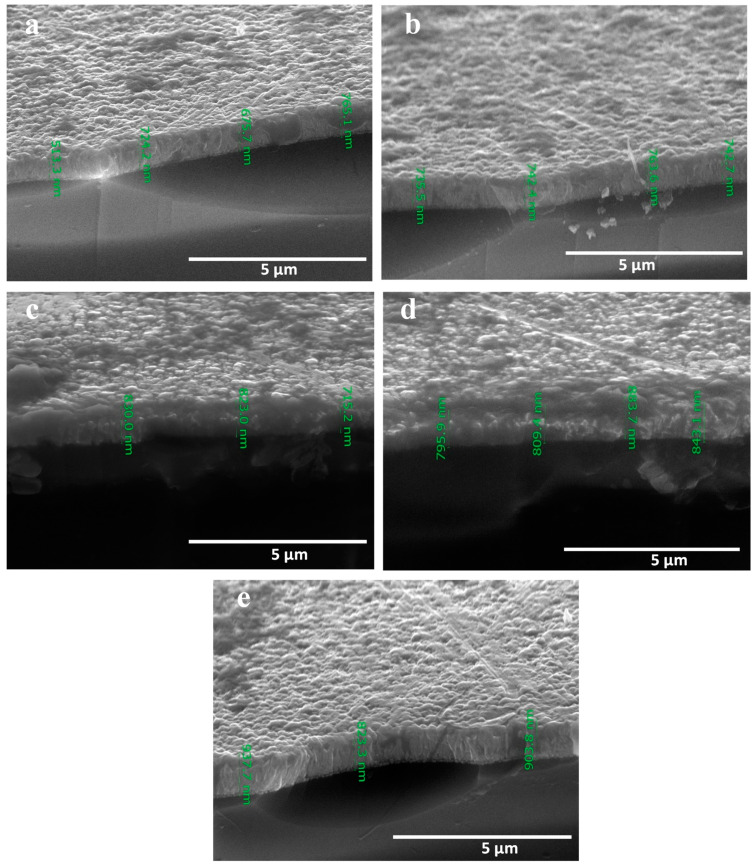
Cross-section SEM images of CdS thin films deposited at different temperatures (**a**) 50 °C (**b**) 100 °C (**c**) 150 °C (**d**) 200 °C, and (**e**) 250 °C.

Figure 4 represents top-view SEM images taken to investigate the morphology of surfaces for CdS thin films which were deposited on FTO substrates at various temperatures (50, 100°, 150, 200, and 250 °C). The surface of the deposited thin films seems to be homogenous, uniform, dense at the surface of the substrates, and free of cracks. The CdS nanocrystals’ morphology varied where the deposited thin films had semi-spherical particles. Along with corresponding with the XRD results, the SEM study also provided further experimental support. As can be observed from Figure 4a–e, as the growth temperature was raised, the size of the crystallites increased, and the crystals took the shape of pyramids, triangles, and spheres. To evaluate the thickness of the film, the cross-section SEM images were measured as presented in Figure 5. It is clear from Figure 5a–e that the CdS formed in thin films with thicknesses of 721, 745, 789, 832, and 887 nm for growth temperatures of 50, 100, 150, 200, and 250 °C, respectively. The increase in film thickness with the rising growth temperature may be attributed to the thermal motion of particles which makes them agglomerate with each other.

### 3.4. Photoluminescence (PL) Analysis

**Figure 6:**
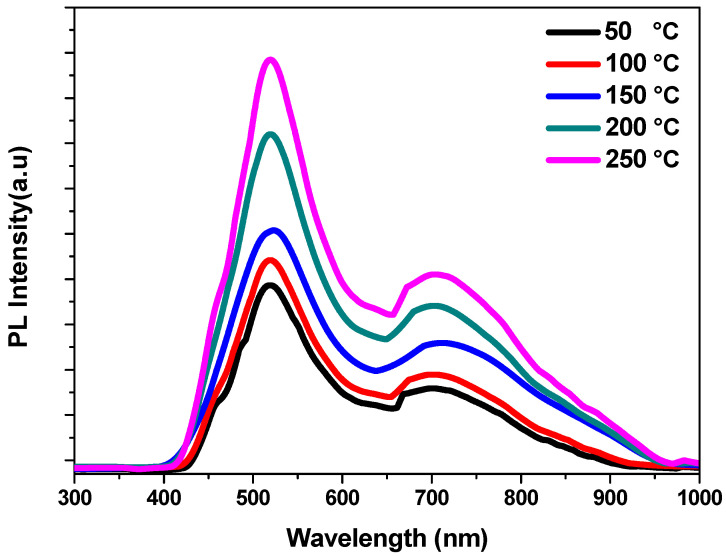
PL spectra of deposited CdS thin films at various temperatures.

Measurements of photoluminescence are used to examine the recombination process of charge carriers [36]. Figure 6 shows the PL spectra of the fabricated CdS thin films grown at various temperatures which were measured at room temperature using an excitation wavelength of 325 nm and with a range of wavelengths between 300 and 1100 nm. All the CdS thin film spectra show two peaks of luminescence that were found to be centered at 520 nm and 705 nm. Band edge emission is linked to the prominent peak at 520 nm, which may be caused by the recombination of free excitons [37]. The peak at 705 nm occurred due to surface-related defects [38]. The move of donors between S-vacancies and S-interstitials produced the green emission peak (520 nm) [39]. The transfer of boundary electrons from surface states to the valence band could be the responsible for the presence of the red emission peak (705 nm) [40]. The increase in crystallite size could be responsible for differences in intensity [41]. Additionally, it appears that the peak’s sharpness and intensity increase with temperature, indicating that sulfur vacancies may be increased with increasing temperatures [42].

### 3.5. Optical Analysis

**Figure 7:**
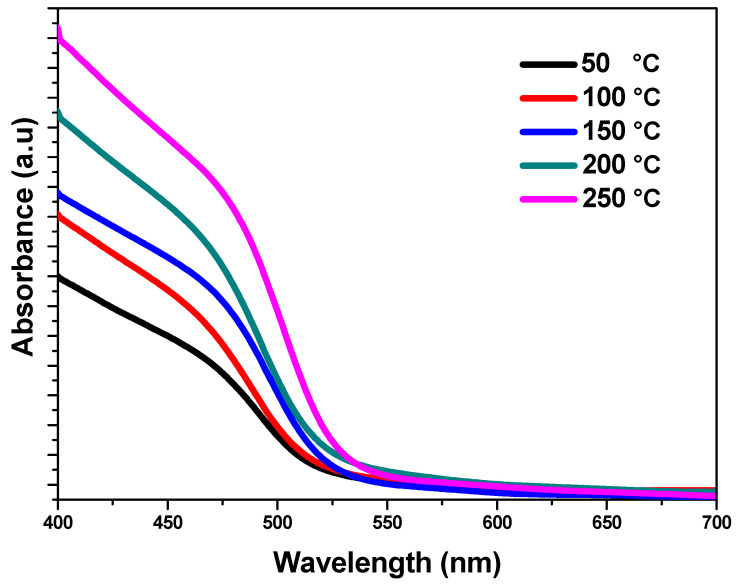
UV–Vis absorption spectra of CdS films at various temperatures.

**Figure 8:**
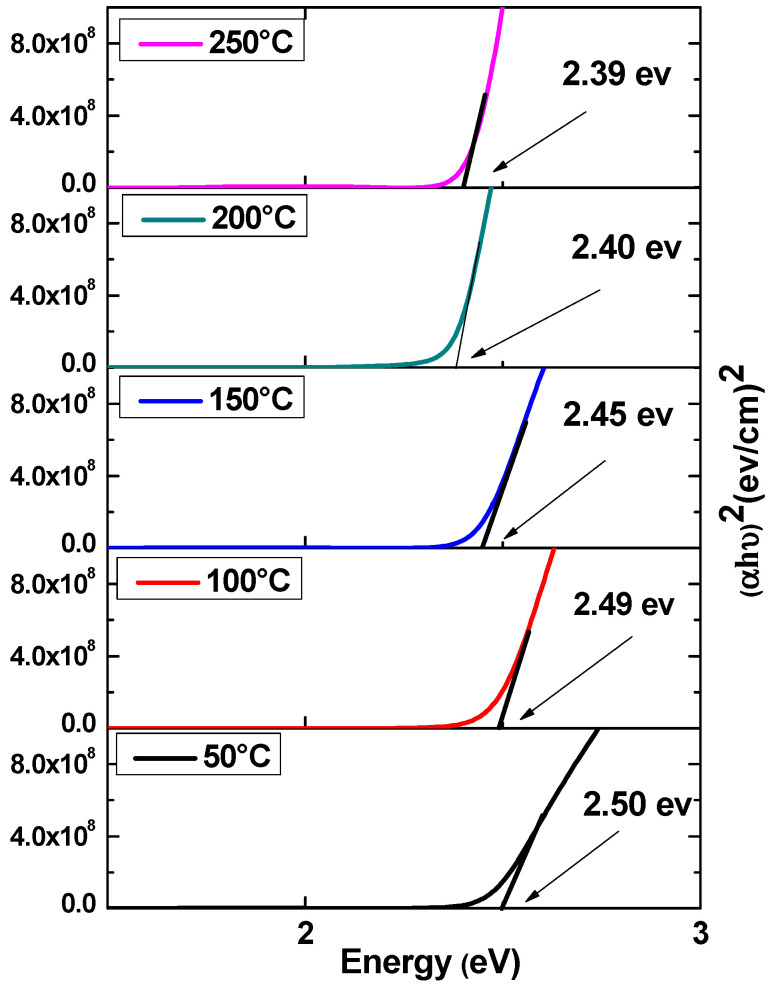
The band gap of CdS thin films at various temperatures.

The optical characteristics of the fabricated CdS thin films, including absorption and bandgap value, were investigated by employing UV–Vis spectroscopy with a wavelength range from 300 to 900 nm. The optical absorption curves and Tauc plots of CdS thin films at various temperatures arrived at via the hydrothermal technique are shown in Figure 7 and Figure 8. The absorption-induced transfer of electrons from the valence band to the conduction band, the UV–Vis absorption characteristic peak of these samples, occurred between 500 and 517 nm and was appointed to the intrinsic optical bandgap of the CdS. On the other hand, when the temperature increased the absorption edge shifted to a wider zone of wavelength, indicating that the bandgap of the formed CdS films declined with a decrease in substrate temperature. At the point where the temperature rose, a sharp rise in light absorption is observed in the visible region. As the absorption level in the 250 °C sample was high, it may have absorbed more photons, which might have enhanced the synthesized product’s photo-sensing activity [41]. The samples’ bandgap energy (Eg) was calculated using Tauc’s relation as the following equation [43,44].

(4) $${\left(\alpha h\upsilon \right)}^{2}=A(h\upsilon -\mathrm{Eg})$$

**Figure 9:**
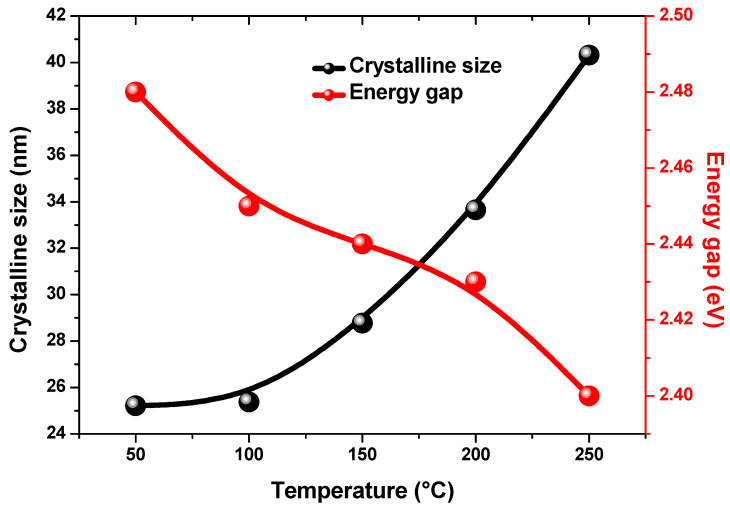
Variation in crystalline size and energy gap for grown CdS films.

Here, α is the absorption coefficient, A is a constant, (*hν*) is the photon energy, h is the Plank’s constant and Eg is the optical band gap. The optical band gap of all CdS thin films is determined by Tauc’s relation plots as presented in Figure 8. The predicted band gap is found to be 2.50, 2.49, 2.45, 2.40, and 2.39 eV for the thin films deposited at 50, 100, 150, 200, and 250 °C, respectively. The calculated optical band gaps were found to show good agreement with previous research that has been published [28,45]. It was found that the optical band gap of the CdS thin films reduced slightly with rises in temperature as shown in Figure 9. This is probably because the crystallite size increased [46].

### 3.6. Photocurrent Measurements

**Figure 10:**
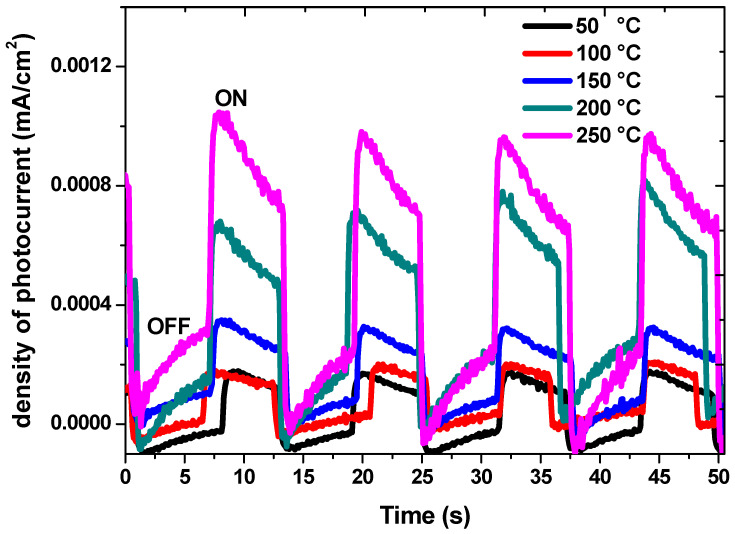
Photocurrent measurements of CdS thin films at different temperatures.

Figure 10 shows the cathodic photocurrent for the fabricated CdS thin films (with an active surface area for the photoelectrode of A = 1 × 1 cm^2^) under the illumination of a 200-watt Halogen and Tungsten lamp where Na_2_SO_4_ solution was used as a supporting electrolyte. The positive photogenerated current values indicate that the CdS samples were n-type semiconductors. The explanation for this could be that when the samples were excited by light, electrons were produced and moved from the electrolyte to the film, and therefore extra negative charges were generated at the semiconductor surface and, consequently, upward photocurrents appeared [47]. It is noticeable that the density of photocurrent under dark conditions (OFF state) is close to 0.00007 mA/cm^2^. Under illumination (ON state), the density of photocurrent increased dramatically with values of 0.00017, 0.0002, 0.00035, 0.00056, and 0.001 mA/cm^2^ for the formed CdS thin films at 50, 100, 150, 200, and 250 °C, respectively. The improvement in charge carrier transfer and the subsequent increase in electron–hole pair collections might have been responsible for the increase in the density of photocurrent, which increased from 0.00017 to approximately 0.001 mA/cm^2^. This also suggests that the reduction in recombination centers is the cause of the rising photocurrent. The conversion of recombination centers into trapping centers explains this decrease in general. The number of ground states is reduced as a result of the higher temperature’s tendency to compress the hole and electron fermi levels together [48]. Moreover, the dark current for the samples deposited at higher temperatures is greater than that of those deposited at lower temperatures, and this could be attributed to the capacitance of the thin films. In such cases, when samples are illuminated, more charge carriers produce current, and with the lamp off, the charge carriers’ lifetime decay will be longer which produces a higher dark current. From this point of view, the samples deposited at higher temperatures will be more sensitive and efficient as photodetectors. These results show good agreement with the PL measurements.

### 3.7. Mott–Schottky and Electrochemical Impedance Spectroscopy (EIS) Analysis

#### 3.7.1. EIS Measurements

**Figure 11:**
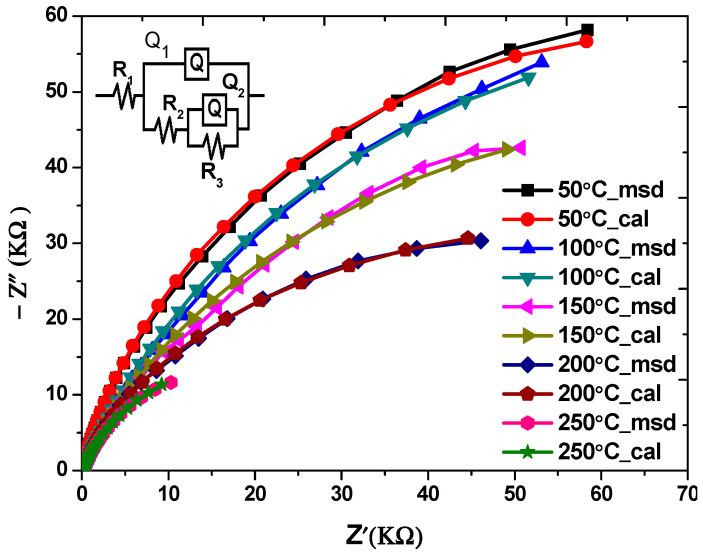
The Nyquist plots of CdS films at various temperatures.

The measurement of electrochemical impedance spectra (EIS) is a useful technique for examining the transfer of charge at the semiconductor/electrolyte interface [49]. These results were obtained at a potential of 1.0 V and a frequency in the range of 10^4^ Hz in 0.5 M Na_2_SO_4_ solutions. Figure 11 shows the Nyquist diagram of CdS thin films deposited. This shows that when the growth temperature increased, the charge transfer resistance (RCT) values dropped. The equivalent electrical circuit model R(Q(R(QR))) was used by the ZSimpWin program to match the Nyquist plot (Z imaginary (*y*-axis) vs. Z real (*x*-axis)) for the CdS thin films. In this model, R_1_ is the solution resistance, R_3_ is the charge transfer resistance, the surface film properties are related to Q_1_ and R_2,_ Q is the constant phase element (CPE), and the nonideal double layer capacity is Q_2_ [50]. Previous research has shown that the diameter of the semicircle is equal to the electron transfer resistance, something which happened because of the heterogeneities at the surfaces of the thin film and the electrolyte and which is therefore associated with the charge transfer at the photoelectrode surface [51]. 

According to the results of the investigation, the realization that all of the semicircles begin at the same point indicates that the electrolyte’s resistance is unchanged [52]. The Nyquist semicircle’s diameter decreased significantly as the temperature of preparation increased, pointing to an increase in the transfer of the photogenerated electron–hole pairs and a decrease in charge recombination, which means that the ability of the electrode to transfer charge is better when the diameter is small [53,54]. 

#### 3.7.2. Mott–Schottky Measurements

**Figure 12:**
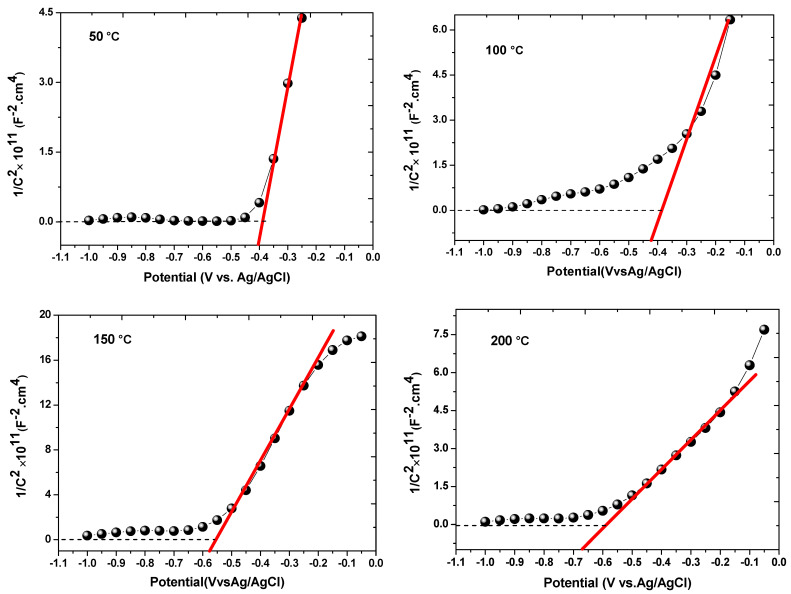

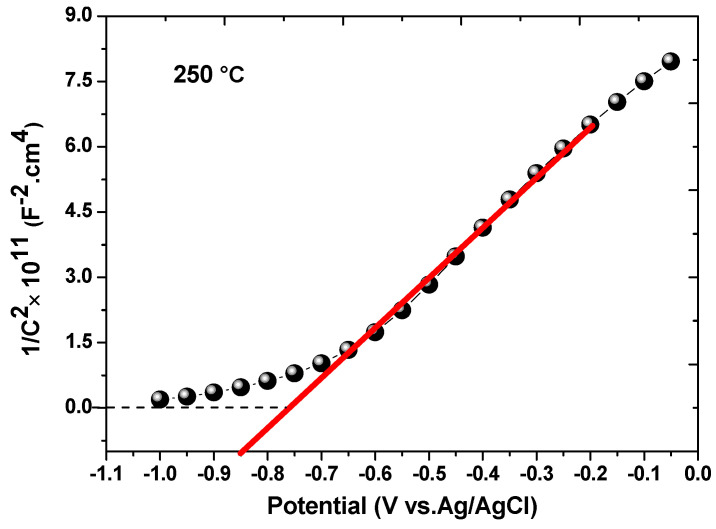
Mott–Schottky plots for CdS thin films with different growth temperatures.

In this work, the Mott–Schottky technique was used to calculate the donor or acceptor concentration and identify the type of deposited films. The Mott–Schottky graph (1/C^2^ vs. V) was investigated using the supporting electrolyte of 0.5 M Na_2_SO_4_ [55]. Figure 12 shows the Mott–Schottky relation for the grown CdS thin films at various temperatures that were measured at a frequency of 1000 Hz. The capacitance was measured in a potential range from −1.1 to 0 V in the cathodic direction. The value of the flat band potential (*V_fb_*) was determined from the x-intercept of the linear portion of the plot given by the graphical representation of the following Mott–Schottky equation [56].

(5) $$\frac{1}{C^{2}}=\frac{2}{-\varepsilon \varepsilon_{0}eA^{2}\;N_{D}\;}\left(V-V_{fb}-\frac{\;kT}{e\;}\right)\;$$

where (*C*) is the semiconductor capacitance, *ε*_0_ is the permittivity of the vacuum (*ε*_0_ = 8.85 × 10^−14^ F/cm), *ε* is the dielectric constant (for CdS = 8.9), *N_D_* is the donor concentration, *e* is the electron charge (*e* = 1.602 × 10^−19^ C), *A* is the active surface area of the photoelectrode (*A* = 1 × 1 cm^2^), *k* is the Boltzmann constant, *T* represents temperature in Kelvin, *V* is the applied potential, and *V_fb_* is the flat band potential [57,58]. The positive slope on the linear (1/C^2^ vs. V) graph shows that CdS has n-type semiconductor properties [59]. The value of *V_fb_* is obtained by extrapolating the Mott–Schottky curve along the x-axis where *N_D_* values are calculated from the following equation [51,60,61]

(6) $$\;\;N_{D}=\frac{2}{-\varepsilon \varepsilon_{0}eA^{2}\;S\;}$$

where *S* is the Mott–Schottky curve slope. The calculated value of *V_fb_* rises from −0.43 to −0.85 V with an increase in growth temperature from 50 °C to 250 °C (Table 2). The calculated value of donor density is increased from 4.41 × 10^18^ to 15.86 × 10^18^ cm^−3^, indicating that sulfur vacancies may be increased with increasing temperature [42].

## 4. Conclusions

Polycrystalline CdS thin films were successfully fabricated on FTO substrates at various temperatures using the hydrothermal method for 2 h. The CdS films were formed in a cubic structure with a preferred (111) orientation, with crystal sizes ranging from 25 to 40 nm. SEM images indicated denseness and good substrate adhesion for the fabricated CdS thin films. Two peaks at 520 and 705 nm were found to be typical for CdS thin films, and could be attributed to the transition between S-vacancies and S-interstitials as well as to the movement of bound electrons from surface states into the valence band. The optical absorption edge around 510 nm indicated the generation of CdS thin films. All thin films were found to have a direct band gap with values which varied from 2.50 to 2.39 eV and which decreased when the growth temperature was raised. All the thin films indicated positive photocurrents which suggested the formation of samples as n-type semiconductors. Resistivity to charge transfer (RCT), as shown by EIS, declined in line with temperatures, reaching its lowest point at 250 °C. Mott–Schottky studies revealed that the flat band potential and donor density changed with temperature, varying with increasing growth temperature from −0.39 to −0.76 V and from 4.41 × 10^18^ to 15.86 × 10^18^ cm^−3^, respectively. Our conclusions reveal that fabricated cubic CdS thin films with such properties will decrease mismatches with other semiconductors used for producing optoelectronic applications such as solar cells, biosensors, and supercapacitors.

## Figures and Tables

**Table 1:** Structural properties of fabricated CdS thin films.

*Temperature (* *°C)*	*2* *θ* *(Degree)*	*FWHM (Degree)*	*Crystallite Size (D) (nm)*	*Micro Strain (ε)* *Lines^−2^ m^4^*	*Dislocation Density (δ) ×10^−4^*	*Energy Band Gap E(g) (eV)*
50	26.48	0.32	25.21	0.34	15.73	2.50
100	26.45	0.31	25.37	0.34	15.53	2.49
150	26.48	0.28	29.77	0.30	12.08	2.45
200	26.45	0.24	33.64	0.26	8.83	2.40
250	26.46	0.20	40.31	0.21	6.15	2.39

**Table 2:** Values of flat band potentials and carrier densities of n-CdS thin films based on growth temperature.

Temperature (°C)	Donner Density (*N_D_*) (cm^−3^)	*V_fb_* (V vs. Ag/AgCl)
50	4.41 × 10^18^	−0.39
100	6.34 × 10^18^	−0.40
150	7.18 × 10^18^	−0.55
200	13.7 × 10^18^	−0.59
250	15.8 × 10^18^	−0.76

## Data Availability

Data available on request.
